# The protective effect of Edaravone against acute renocardiac syndrome in a kidney ischemia-reperfusion model

**DOI:** 10.34172/jcvtr.33077

**Published:** 2024-12-23

**Authors:** Yasin Bagheri, Mahshid Dehghan, Seyyedeh Mina Hejazian, Mohammadreza Ardalan, Sepideh Zununi Vahed, Bahram Niknafs

**Affiliations:** ^1^Kidney Research Center, Tabriz University of Medical Sciences, Tabriz, Iran; ^2^Faculty of Medicine, Tabriz University of Medical Sciences, Tabriz, Iran

**Keywords:** Edaravone, Ischemia-reperfusion, Antioxidants, Cardiorenal syndrome

## Abstract

**Introduction:**

Acute kidney injury (AKI) is a common clinical occurrence causing high mortality and morbidity. In acute renocardiac syndrome, AKI leads to acute cardiac injury or/and dysfunction. This study aimed to investigate the antioxidative effects of Edaravone on cardiac tissues following the induction of renal ischemia-reperfusion injury (IRI) in rats.

**Methods:**

Twenty-four male Wistar rats were randomly divided into four groups: IR+Edaravone, Edaravone, IR, and Sham groups (six rats per group). Non-traumatic clamps were used to stop the artery and vein blood flow of the left kidney in rats of the IR groups for 45 minutes. Thirty minutes before ischemia induction, Edaravone (3 mg/kg) was injected intraperitoneally in the IR+Edaravone group. Cardiac samples were subjected to biochemical analyses.

**Results:**

The Results showed a significant increase in the enzymatic activity of glutathione peroxidase (*P*=0.01), catalase (*P*=0.03), and superoxide dismutase (*P*=0.02), and the levels of glutathione (*P*=0.012), and total antioxidant capacity (*P*<0.001) in the IR+Edaravone group in comparison to the IR group. Moreover, the total antioxidant capacity of the heart was increased in the Edaravone group compared to the control and IR groups (*P*<0.001), indicating the safety of the drug.

**Conclusion:**

The results can reveal important insights into the protective effects of Edaravone against acute renocardiac syndrome.

## Introduction

 Ischemic acute kidney injury (AKI) is likely to occur following organ transplantation, sepsis, and myocardial infarction. It is prevalent among almost two-thirds of ICU (intensive care unit) and one-fifth of hospitalized patients.^1,2^ Renal ischemia/reperfusion injury (IRI) initiates a cascade of biochemical and molecular alterations that can induce apoptosis, inflammation, and oxidative stress. It has become evident that a significant portion of the heightened AKI-related mortality is attributed to remote organ complications. Individuals with kidney diseases have a higher possibility of developing cardiovascular diseases,^3^ the main causes of death among patients with AKI.^4,5^ The connection between the kidneys and heart has been identified as a clinical condition known as cardiorenal syndrome (CRS).

 Acute renocardiac syndrome is a specific subclassification of CRS (CRS type 3) in which an episode of AKI triggers the emergence of acute cardiac injury and (or) dysfunction. Previous studies have shown that renal IRI results in heart dysfunction by increasing inflammatory factors.^6,7^ Moreover, the renal IRI triggers oxidative stress within the heart, as evidenced by elevated levels of lipid peroxidation and reduced antioxidant defense. Oxidative stress involves the overproduction of reactive oxygen species (ROS) and their buildup due to the impairment of endogenous antioxidant capacity.^8,9^

 Free radical scavengers are currently being used to mitigate the damage caused by oxidative stress in the myocardium.^6,10^ Edaravone, a compound known as 3-methyl-1-phenyl-2-pyrazolin-5-one, acts as a free radical scavenger. In addition to its scavenging properties, experimental studies have shown that Edaravone also possesses anti-necrotic, anti-apoptotic, and anti-inflammatory effects in cardiovascular diseases and stroke. These effects further validate its protective capabilities in the brain, heart, kidney, and blood vessels.^11-14^ Moreover, the results of a clinical trial demonstrated that the administration of Edaravone before myocardial reperfusion was associated with reduced enzymatic infarctions and improved clinical prognosis.^15^

 Since there is no evidence about the cardioprotective effects of Edaravone on CRC type 3, this study aimed to investigate its impact on the endogenous antioxidant capacity of the heart after renal IR induction in an experimental rat model. Antioxidant enzyme activity [i.e. glutathione peroxidase (GPx), superoxide dismutase (SOD), and catalase (CAT)], and levels of total antioxidant capacity (TAC) and glutathione (GSH) along with malondialdehyde levels (MDA) were evaluated in heart tissue after kidney IRI.

## Materials and Methods

###  Study design 

 Twenty-four male Wistar rats, weighing 230 ± 15 g, were included (Pasteur Institute of Iran, Tehran, Iran). Ten days prior to experiments, rats were housed on a 12-hour light and 12-hour dark cycle with controlled room temperatures (22 ± 2°C), humidity (40-70%), and free access to food and water. Then, the rats were divided randomly into four groups including Sham, IR, Edaravone, and IR + Edaravone pretreated rats (6 rats in each group). A single dose of 3 mg/kg Edaravone diluted in saline (3 mg/ml) was intraperitoneally injected into groups 3 and 4 of rats one hour before IR induction.^16-18^

###  Kidney IR induction

 Intraperitoneal injection of Ketamine (90 mg/kg) and xylazine (10 mg/kg) was performed simultaneously for anesthetizing rats. Induction of renal ischemia-reperfusion was done as explained in our previous studies.^19,20^ Briefly, after confirming the absence of a corneal reflex, a middle skin incision was performed for kidney vascular manipulation. Non-traumatic clamps were used to stop the artery and vein blood ﬂow of the left kidney in rats of the IR groups for 45 minutes. The manipulation of rats in the Sham group was without clamping. After confirming ischemia in the kidney by observing paled-colored kidneys, renal perfusion was reestablished by removing forceps. A heating pad was used during the surgical procedure to maintain the body temperature of the animals at 37 °C. Finally, the abdominal cavity of rats was stitched and they were returned to their cage. Six hours later reperfusion, intraperitoneal injection of Thiopental sodium (200 mg/kg) was administered for euthanizing rats and heart tissue was isolated.^19,20^

###  Biochemical analysis

 The total oxidant/antioxidant levels and related enzyme activity of heart tissue were determined by measuring levels of TAC, and GSH and antioxidant enzyme activities of SOD, and CAT in the heart according to the protocol of Zellbio kits (Biocore, Germany).^21^ The activity of GPX was colorimetrically evaluated using a microplate reader at 412 nm. Besides, lipid peroxidation was assessed by measuring MDA levels via the ZellBio MDA assay Kit (Biocore Diagnostik, Germany).

###  Statistical analysis

 Statistical analysis was performed utilizing the SPSS version 23.0 developed by IBM. The assessment of data normality for each variable was carried out through the Shapiro–Wilk test. All data were presented as mean ± standard deviation. Moreover, one-way ANOVA followed by Tukey post-hoc tests were applied to compare the results between the studied groups. A p-value below 0.05 was considered statistically significant.

## Results

 To evaluate the antioxidative effect of Edaravone against cardiac injury after renal IRI, levels/activity of anti-oxidants and MDA were evaluated and compared between the studied groups. In cardiac tissue, the reduction in antioxidants and enhanced production of MDA were revealed in the IR group compared to the Sham group, confirming the development of CRS3, Table 1. Results showed a significant increase in the activity of GPx (10.95 ± 1.55 versus 8.62 ± 0.53, *P* = 0.01), CAT (4.00 ± 0.26 versus 3.06 ± 0.06, *P* = 0.03), and SOD (2.04 ± 0.3 versus 1.47 ± 0.12, *P* = 0.02) enzymes in the IR + Edaravone pretreatment group in comparison to the IR group. Moreover, the levels of GSH (40.60 ± 3.40 versus 31.12 ± 1.6, *P* = 0.012), and TAC (1.90 ± 0.16 versus 1.38 ± 0.16, *P* < 0.001) increased significantly in the IR + Edaravone pretreatment group in comparison to the IR group. A decrease in cardiac levels of MDA (0.45 ± 0.14 versus 0.59 ± 0.13) was observed in the Edaravone pre-treated IR group compared to the IR group; however, it was not statistically significant (*P* = 0.07). To evaluate the safety and direct effect of Edaravone on the heart, levels of oxidant/anti-oxidant factors were evaluated in the Edaravone treatment group and were compared to the Sham and IR groups. Results showed a significant increase in the activity of TAC (*P* < 0.001), CAT (*P* = 0.02), GSH (*P* = 0.006), and SOD (*P* = 0.003) levels in the Edaravone group compared to the IR group, indicating that Edaravane elevated the antioxidant activities/levels in the heart tissue. Cardiac levels of the MDA and GPx between the Edaravone and the IR groups were not significantly different (Table 1).

**Table 1:** Oxidative activity of heart tissue in the studied groups

**Groups**	**SOD (U/mg protein)**	**MDA (nmol/mg protein)**	**GPx (U/mg protein)**	**TAC (mmol/mg protein)**	**GSH (mmol/mg protein)**	**Catalase (U/mg protein)**
Sham (n = 6)	1.93 ± 0.11	0.51 ± 0.12	9.32 ± 0.82	1.88 ± 0.09	36.40 ± 4.11	3.12 ± 0.17
IR (n = 6)	1.47 ± 0.12^*^	0.59 ± 0.13	8.62 ± 0.53	1.38 ± 0.16^**^	31.12 ± 1.69	3.06 ± 0.06
Edaravone (n = 6)	2.09 ± 0.33^##^	0.41 ± 0.12	10.60 ± 1.53	2.02 ± 0.25^###^	43.04 ± 5.80^##^	4.04 ± 0.49^#^
IR + Edaravone (n = 6)	2.04 ± 0.31^#^	0.45 ± 0.14	10.95 ± 1.55^#^	1.90 ± 0.16^##^	40.60 ± 3.40^#^	4.00 ± 0.26^#^

GPx: glutathione peroxidase, GSH: glutathione, IR: ischemia/reperfusion, MDA: malondialdehyde, SOD: superoxide dismutase, TAC: total antioxidant capacity. The obtained results are represented as mean ± standard deviation. * and **: indicating a remarkable difference (*P* < 0.05 and < 0.01, respectively) between the IR group and the Sham group. #, ##, and ### indicating a remarkable difference (*P* < 0.05, < 0.01, and < 0.001, respectively) in comparison to the IR group.

## Discussion

 The present experimental investigation demonstrated that pre-treatment with Edaravone before the initiation of kidney IRI resulted in a notable enhancement in the levels and activities of antioxidants in the heart, suggesting the protective impact of this medication against acute renocardiac syndrome.

 Numerous pharmacological agents have been identified as potential preventive measures against the detrimental effects of free oxygen radicals, which play a crucial role in IR injury. However, despite their potential, most of these agents have not been widely adopted in clinical practice. In contrast, Edaravone, a medication known for its neurovascular protective effects, has been successfully utilized in patients with acute ischemic stroke in Japan since April 2001. Edaravone exhibits various beneficial properties, including antiapoptotic, free radical scavenging, anti-necrotic, and anti-inflammatory activities. Edaravone has various cardioprotective effects, making it an effective therapeutic option for cardiovascular diseases, stroke, and the protection of vital organs such as the heart, blood vessels, kidneys, and brain.^4,14^ Previous studies have verified that Edaravone prevents myocardial injury and improves cardiac function through scavenging free radical oxygen and oxidative stress,^22,23^ decreases the infarct size by eliminating the apoptotic cells,^24^ and prevents cardiac arrhythmias.^25^

 Malondialdehyde is a byproduct of the peroxidation of fatty acids and the occurrence of oxidative stress within cells. The surge in free ROS resulting from IRI leads to an increase in the MDA levels. Our results showed a decrease in cardiac levels of MDA in the Edaravone pre-treated IR group compared to the IR group; however, it was not statistically significant. Yagi et al observed a notable reduction in MDA levels within the blood samples of rats following treatment with Edaravone. The researchers assessed the impact of Edaravone on cardiac function by examining lipid peroxidation after inducing cardiac ischemia-reperfusion in rat hearts. They administered an intravenous injection of 3 mg/kg of Edaravone after 2-3 minutes of cardiac IR, achieved by ligating the coronary artery. Based on their findings, the Edaravone group exhibited lower MDA serum levels compared to both the sham and IR groups of rats.^25^ Zhang et al conducted a study to assess the cardioprotective properties of Edaravone in the context of IRI in the cardiac myocardium. One minute before IRI induction through ligation of the left anterior descending coronary artery, 3 and 10 mg/kg of Edaravone was administered. In contrast to our findings, their results suggest a notable reduction in the levels of MDA in the blood samples following Edaravone treatment.^26^ The dissimilarity in the findings of our study compared to previous studies could potentially be attributed to variances in the dosage of administered Edaravone and the extended time interval between Edaravone treatment and IRI, and also measuring tissue versus blood levels of MDA.

 Cellular capacity and potential against oxidative stress rely heavily on TAC. TAC serves as the initial step in evaluating the body’s ability to withstand oxidative stress and balance between levels of oxidative and antioxidative factors. The results of this study indicate that Edaravone effectively enhances the TAC levels in cardiac tissue following renal IRI. In a similar vein, Liu et al conducted a study to assess the renoprotective properties of Edaravone in rats with AKI induced by sepsis. Their findings revealed that administering 3 mg/kg of Edaravone not only elevates the TAC level but also mitigates oxidative stress-induced damage and restores antioxidant alterations in mitochondria.^27^ Ergenoglu and colleagues conducted a study to assess the impact of Edaravone on improving ovarian IRI induced by pneumoperitoneum in rats. Their findings revealed that administering 6 and 12 mg/kg of Edaravone resulted in a significant elevation of oxidative stress markers, specifically TAC.^28^ Based on the results of previous investigations, it can be concluded that Edaravone treatment effectively mitigates oxidative stress induced by IRI by enhancing TAC levels.

 Superoxide dismutase plays a crucial role in facilitating the conversion of superoxide, a byproduct of oxygen, into either regular oxygen or hydrogen peroxide. In this study, Edaravone administration effectively increased SOD activity and protected cardiac tissue from oxidative stress caused by renal IRI. Zhang et al also found similar results regarding SOD elevation.^26^ Consistent with our findings, the study of Yamawaki et al confirmed that Edaravone effectively elevated the SOD activity while reducing the presence of ROS. Consequently, this direct intervention exhibited a protective effect on the cardiac cells, mitigating the impact of kidney IRI.^29^ The accumulation of hydrogen peroxide, the end product of SOD, can be toxic to cells and body tissues. In the presence of Fe^2+^, it can convert into highly reactive hydroxyl radicals that can cause damage. However, to counteract this, catalase acts as an antioxidant and converts hydrogen peroxide into water and oxygen and therefore prevents the damage caused by free radicals. It is important to note that catalase is not present in the mitochondria, so the conversion of hydrogen peroxide to water and oxygen is carried out by GPX in the presence of GSH. The findings of the present study indicate that the administration of Edaravone leads to a significant enhancement in the activity of CAT, GSH, and GPX in the cardiac tissue. In a similar vein, Quamrul et al induced an infarct in rabbit hearts and subsequently treated them with Edaravone at doses of 1, 3, and 10 mg/kg. Their results demonstrated a significant elevation in the antioxidant levels of SOD, CAT, GSH, and GPx following Edaravone treatment.^30^ Yu et al conducted a study to determine the impact of combining mild hypothermia with Edaravone on cerebral IRI. The researchers administered a dosage of 3 mg/kg of Edaravone to the rats following the induction of occlusion in the middle cerebral artery. The outcomes of their investigation revealed a noteworthy increase in the levels of SOD, CAT, GSH, and GPX. Consistent with our findings, the study also observed no significant reduction in the levels of MDA after the administration of Edaravone.^31^ İnce et al examined the renoprotective properties of Edaravone following limb IRI. They administered a dosage of 6 m/kg of Edaravone 30 minutes after clamping the abdominal aorta and inducing limb IRI. The findings of their study revealed a noteworthy elevation in serum SOD levels, along with a considerable reduction in both serum and tissue MDA levels. However, it is important to note that their study did not observe any increase in GPx levels.^32^

 The rise in SOD and CAT antioxidant enzyme levels could potentially be attributed to a reduction in free radicals as a result of the Edaravone treatment. Furthermore, the increase and enhancement in GPx activity may be attributed to an increase in GSH levels following Edaravone treatment. As a result, the prevention of ROS generation leads to an increase in TAC capacity by maintaining a balance between oxidant and antioxidant factors. In conclusion, the increase in levels and activity of endogenous antioxidant enzymes, along with the improvement in TAC, may be attributed to the potential suppressive effect of Edaravone against free radicals.

 This study had some unpredicted limitations. Previous studies administered multiple injections of Edaravone at specific intervals and compared outcomes, but this study only administered one dose one hour before renal ischemia. It is necessary to evaluate different diluted doses of Edaravone at different times (before, during, and after ischemia) and compare the results. Additional research is recommended on other molecular mechanisms of Edaravone in cardiac tissue after kidney IRI, such as apoptosis, mitochondrial function, inflammation, etc. It is also advised to investigate the combined effects of Edaravone with other antioxidant drugs.

 While Edaravone has a strong capacity to neutralize free radicals, its poor stability, solubility, and short circulation half-life have restricted its therapeutic efficacy. Nanoformulations of Edaravone can enhance its efficacy and address the aforementioned limitations.^33-35^ These nanoformulations include self-nanomicellizing solid dispersions, lipid-based nanosystems, and nanogels. The development of these nanoformulations aims to improve the oral bioavailability, stability, dissolution, and permeability of Edaravone in different diseases.^33-36^

## Conclusion

 This study indicated that administering a single dose of Edaravone through intraperitoneal injection could increase the antioxidant activity of heart tissue after renal IRI. The results of the current research have revealed important insights into the protective effects of Edaravone on acute renocardiac syndrome (AKI-induced cardiac injury). It is imperative to conduct further clinical studies are imperative to explore alternative molecular pathways of Edaravone within cardiac tissue prior to its utilization as an antioxidative agent in cardiovascular disorders.

## Competing Interests

 The authors declared that they had no conflict of interest.

## Ethical Approval

 This study was approved by the ethical committee of Tabriz University of Medical Sciences (Ethical code: IR.TBZMED.AEC.1402.082). The study was performed according to the declaration of Helsinki and all the experimental animals received humane care.
